# THE CONTEXTUAL EXPERIENCES OF SOCIAL CAPITAL AMONG OLDER GRANDPARENTS RAISING ADOLESCENT GRANDCHILDREN

**DOI:** 10.1093/geroni/igz038.1049

**Published:** 2019-11-08

**Authors:** Tina L Peterson

**Affiliations:** University of Oklahoma, Norman, Oklahoma, United States

## Abstract

Older grandparents raising adolescent grandchildren are an understudied population. Greater understanding is needed of the social capital (e. g. information, emotional support, companionship, practical instrumental support, and influence, power, and control) harnessed by older grandparents raising adolescent grandchildren. This research applied a qualitative, phenomenological approach to explore social capital among older grandparents. Face-to-face interviews were conducted with 19 grandparent caregivers ranging in age from 55 to 88 years. Eligibility criteria were: primary caregiver for a grandchild 12 years or older; grandchild resides in home at least 3 days; grandparent 40 or older and resides in Oklahoma, Alabama, or Kentucky. Participants were recruited by word of mouth, newspapers, and flyers. Grandparents responded to a question prompt, “I am going to ask you some questions about your support system to assist with your concerns about your older grandchild.” Interviews were conducted in public places, audiotaped, and transcribed verbatim. Data were analyzed using a question analysis approach to sort responses into matrices, develop memos, and identify themes. Most older grandparents were female (84.2%), Caucasian (52.6%), married (57.9%), and never attended a support group (68.4%). One overarching theme from these older grandparent caregivers is access to social capital exists on a continuum. Participants’ experiences with social capital pertained to family proximity, evolving perception of friends, limited or no social engagement with neighbors, dynamics of church attendance and size, and familiarity with community resources. These older grandparents raising adolescents shared positive reactions to select social capital with some types emerging as less important or underdeveloped.

